# MRI-guided radiotherapy of the SK-N-SH neuroblastoma xenograft model using a small animal radiation research platform

**DOI:** 10.1259/bjr.20160427

**Published:** 2017-01

**Authors:** Aurélien Corroyer-Dulmont, Nadia Falzone, Veerle Kersemans, James Thompson, Mark Hill, P Danny Allen, John Beech, Stuart Gilchrist, Paul Kinchesh, Boris Vojnovic, Iain Tullis, Mark N Gaze, Sean Smart, Katherine A Vallis

**Affiliations:** ^1^ CR-UK/MRC Oxford Institute for Radiation Oncology, Department of Oncology, University of Oxford, Oxford, UK; ^2^ University College London Hospitals NHS Foundation Trust, London, UK

## Abstract

**Objective:**

Neuroblastoma has one of the lowest survival rates of all childhood cancers, despite the use of intensive treatment regimens. Preclinical models of neuroblastoma are essential for testing new multimodality protocols, including those that involve radiotherapy (RT). The aim of this study was to develop a robust method for RT planning and tumour response monitoring based on combined MRI and cone-beam CT (CBCT) imaging and to apply it to a widely studied mouse xenograft model of neuroblastoma, SK-N-SH.

**Methods:**

As part of a tumour growth inhibition study, SK-N-SH xenografts were generated in BALB/c nu/nu mice. Mice (*n* = 8) were placed in a printed MR- and CT-compatible plastic cradle, imaged using a 4.7-T MRI scanner and then transferred to a small animal radiation research platform (SARRP) irradiator with on-board CBCT. MRI/CBCT co-registration was performed to enable RT planning using the soft-tissue contrast afforded by MRI prior to delivery of RT (5 Gy). Tumour response was assessed by serial MRI and calliper measurements.

**Results:**

SK-N-SH xenografts formed soft, deformable tumours that could not be differentiated from surrounding normal tissues using CBCT. MR images, which allowed clear delineation of tumours, were successfully co-registered with CBCT images, allowing conformal RT to be delivered. MRI measurements of tumour volume 4 days after RT correlated strongly with length of survival time.

**Conclusion:**

MRI allowed precision RT of SK-N-SH tumours and provided an accurate means of measuring tumour response.

**Advances in knowledge:**

MRI-based RT planning of murine tumours is feasible using an SARRP irradiator.

## INTRODUCTION

Neuroblastoma is the most frequent extracranial tumour in infants and young children. The treatment of high-risk neuroblastoma usually consists of some mix of surgery, chemotherapy, radiotherapy (RT), stem cell transplant, immunotherapy and retinoid therapy.^1^ However, because at 40%, the 4-year overall survival for late-stage neuroblastoma is still low compared with other childhood malignancies, new combination regimens are being developed and tested.^1,2^ In some cases, it is desirable to test investigational protocols in preclinical models of the disease to establish efficacy. Xenograft mouse models are frequently used to study the effects of anticancer drugs and RT *in vivo*, and the SK-N-SH human neuroblastoma model has been widely studied for this purpose.^3^ The aim of this short communication was to present a method for MRI-guided RT of SK-N-SH xenografts and to track post-treatment changes using MRI and using techniques previously developed for the delivery of RT to pancreatic tumours.^4^ This method development study involved eight mice that were a subgroup of animals used in a larger therapy study (to be reported separately).

## METHODS AND MATERIALS

### Cell line

The SK-N-SH human neuroblastoma cell line was obtained from American Type Culture Collections (ATCC, Manassas, VA) and cultured in Dulbecco's Modified Eagle Medium (DMEM: Sigma Aldrich, UK) supplemented with 10% foetal calf serum (InVitrogen, UK), 2-mM glutamine (Sigma–Aldrich, UK) and 100-U ml^−1^ penicillin/streptomycin (InVitrogen, UK).

### Mouse neuroblastoma model

**Figure 1.**
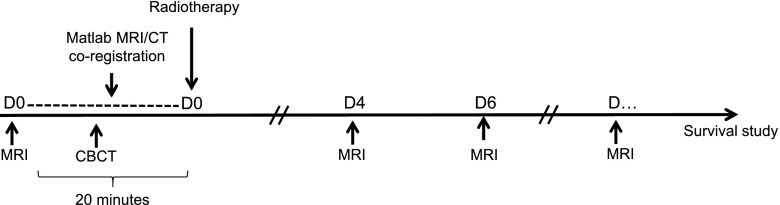
Experimental schema showing the timing in days (D) of imaging events and treatment. CBCT, cone-beam CT; D0, Day 0; D4, Day 4; D6, Day 6.

All animal procedures were carried out in accordance with the UK Animals (Scientific Procedures) Act 1986 and with the local ethics committee approval. In preliminary experiments, the frequency of tumour development following subcutaneous injection of SK-N-SH cells into the flanks of nude athymic mice (BALB/c nu/nu) was found to be low (10%). Therefore, SK-N-SH cells (5 × 10^6^) in 100 µl of matrigel were injected subcutaneously into the flanks of non-obese diabetic/severe combined immunodeficiency mice (20–25 g, 8 weeks old, female, Charles River, UK). In this mouse strain, which is profoundly immunodeficient and is therefore an excellent recipient for engrafted human cells, the success rate of xenograft development was 100%. Tumours were excised and homogenized when they reached a geometric mean diameter of 10 mm (at approximately 5 weeks). Tumour homogenates were then injected into the right flank of recipient BALB/c nu/nu mice (15–20 g, 8 weeks old, female, Charles River, UK). Following this procedure, the proportion of BALB/c nu/nu mice that developed xenograft tumours was 100%. A common method to assess tumour volume is to measure the two longest perpendicular axes in the *x*–*y* plane using callipers and then the depth is assumed to be equivalent to the shortest of the perpendicular axes. However, SK-N-SH xenografts were flat in shape and easily deformable and it was not possible to obtain accurate volume measurements using this method. Therefore, in this study, when using callipers, tumour size was reported as the maximum tumour diameter. Animals were entered into study when their tumour volume reached 500 mm^3^ as measured by MRI. Figure 1 shows a summary of the timing of imaging events and treatment.

### MRI

**Figure 2.**
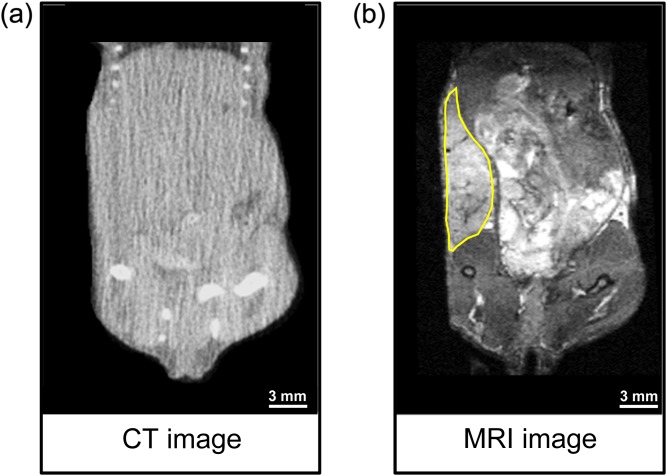
(a) Cone-beam CT (CBCT) and (b) MR whole-body images of a representative SK-N-SH xenograft-bearing mouse. The tumour is outlined in yellow on the MR image but is not visible on the CBCT image.

**Figure 3.**
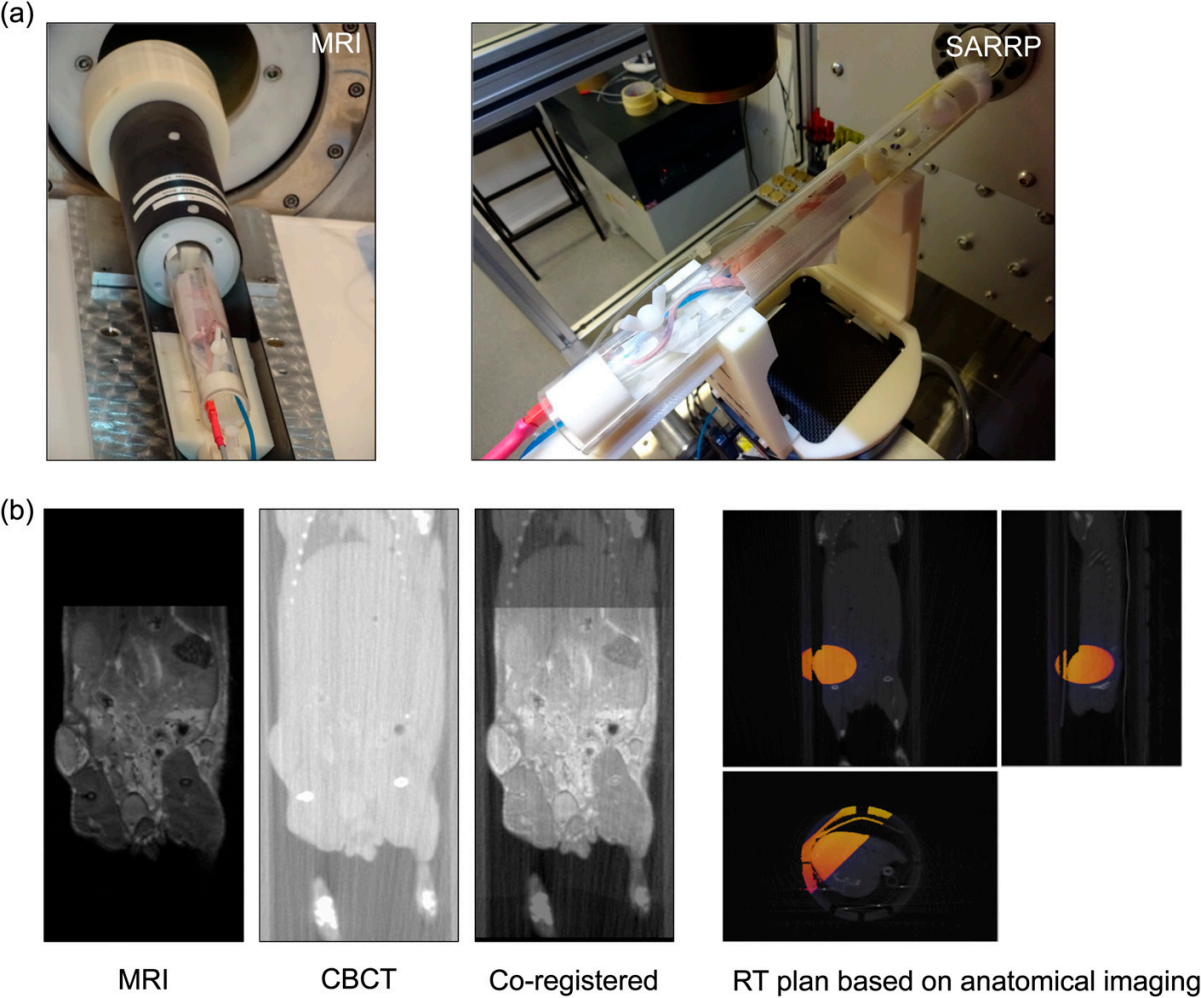
MRI-guided RT protocol: (a) the cradle positions on the MRI scanner (left) and small animal radiation research platform (SARRP) (right). (b) The radiotherapy (RT) protocol involves MRI anatomical whole-body image acquisition, followed by CBCT whole-body acquisition, MATLAB^®^ (MathWorks^®^, Natick, MA) rigid co-registration and target volume planning using anatomical information from MRI and Muriplan software.

SK-N-SH xenografts were not visible in CT images acquired using the on-board small animal radiation research platform (SARRP)-cone-beam CT (CBCT) and so, these images were unsuitable for RT planning (Figure 2a). In contrast, for the same mouse, tumours were clearly seen in MR images (Figure 2b). Therefore, we designed an MR- and CT-compatible plastic cradle suitable for use in the SARRP system (Xstrahl Ltd, Camberley, UK). The cradle design allowed anaesthetic gas delivery, rectal thermometry, electrical heating and respiration monitoring. For all experiments, mice were maintained under anaesthesia: 4% isoflurane for induction, 2% for maintenance in air supplemented with O_2_ (70%/30% v/v). Mice were placed into the cradle in the supine position, allowing maintenance of the same geometry on transfer of animals from the MR to the SARRP-CBCT scanner (Figure 3a). Respiration was monitored using a pressure-sensitive balloon around the abdomen. MRI was performed using a 4.7-T 310-mm horizontal bore varian nuclear MR spectrometer (VNMRS) (Varian Inc., Palo Alto, CA, USA) preclinical imaging system and a 120-mm bore gradient insert with maximum gradient strength 400 mT m^−1^ in all axes, with transmission and reception performed using a 32-mm quadrature birdcage coil (Rapid Biomedical GmbH, Rimpar, Germany). Tumours were detected with a respiratory-gated two-dimensional *T*_2_ weighted sequence (fast spin echo, effective echo time = 22.43 ms, repetition time = 1556.49 ms, slice thickness = 0.33 mm, 58 slices) using slice-projection loop index counter-enabled reacquisition. A respiratory-gated three-dimensional balanced steady-state free-precession sequence was used as an anatomical reference for MRI/CBCT co-registration (field of view 60 × 30 × 30 mm^3^, matrix 256 × 128 × 128 pixels and a 15° degree flip with a 16-μs hard pulse).

### Radiotherapy

Animals were entered into the study when their tumour volume reached 500 mm^3^ (at about 7 weeks after inoculation of SK-N-SH cells) and were randomly assigned to two groups: control (no treatment) or external beam RT. RT treatment (220-kVp X-rays; half-value layer of 0.93 mmCu) was administered using the SARRP irradiator at a dose rate of 2 Gy min^−1^ using a range of custom collimators depending on the tumour geometry. Dosimetry was performed using an EBT3 film (Ashland ISP Advanced Materials, Wayne, NJ) that was calibrated against absolute measurements determined by following the recommendations of the report of the American Association of Physicists in Medicine Task Group 61.^5^ On transfer from the MR scanner to the irradiator, MR images were imported into the SARRP system (Figure 3b). MR images were co-registered with the SARRP-CBCT image using an in-house MATLAB^®^ (MathWorks^®^, Natick, MA, USA) code based on the modality independent neighbourhood descriptor algorithm for multimodal deformable registration.^6^ The resulting MR images were then imported into the Muriplan software (Xstrahl Ltd, Camberly, UK), allowing the target volume to be clearly identified, enabling optimal beam delivery (Figure 3b). A single fraction of 5 Gy was delivered which, based on published data, was predicted to give a robust tumour response in this model.^7^ The time from MRI acquisition to the end of the RT delivery was approximately 20 min. To monitor tumour response to RT, eight mice (three controls; five in RT group) were imaged weekly using MRI. Mice were euthanized when the tumour volume reached 800 mm^3^.

### Image processing and analysis

Image analysis was performed using ImageJ (National Institutes of Health, Bethesda, MD) software. Tumour volume delineation was performed manually on all adjacent *T*_2_ weighted slices. The tumour volume was calculated by multiplying the area of the tumour on each slice by the slice thickness and then adding these together.

### Statistical analyses

All data are presented as mean ± standard deviation. One-way analysis of variance followed by Tukey's *post hoc* test was used to assess differences in tumour volume between groups. A log-rank test was used to compare Kaplan–Meier curves. Statistical analyses were obtained with GraphPad Prism^®^ software (GraphPad Software, Inc., La Jolla, CA).

## RESULTS

**Figure 4.**
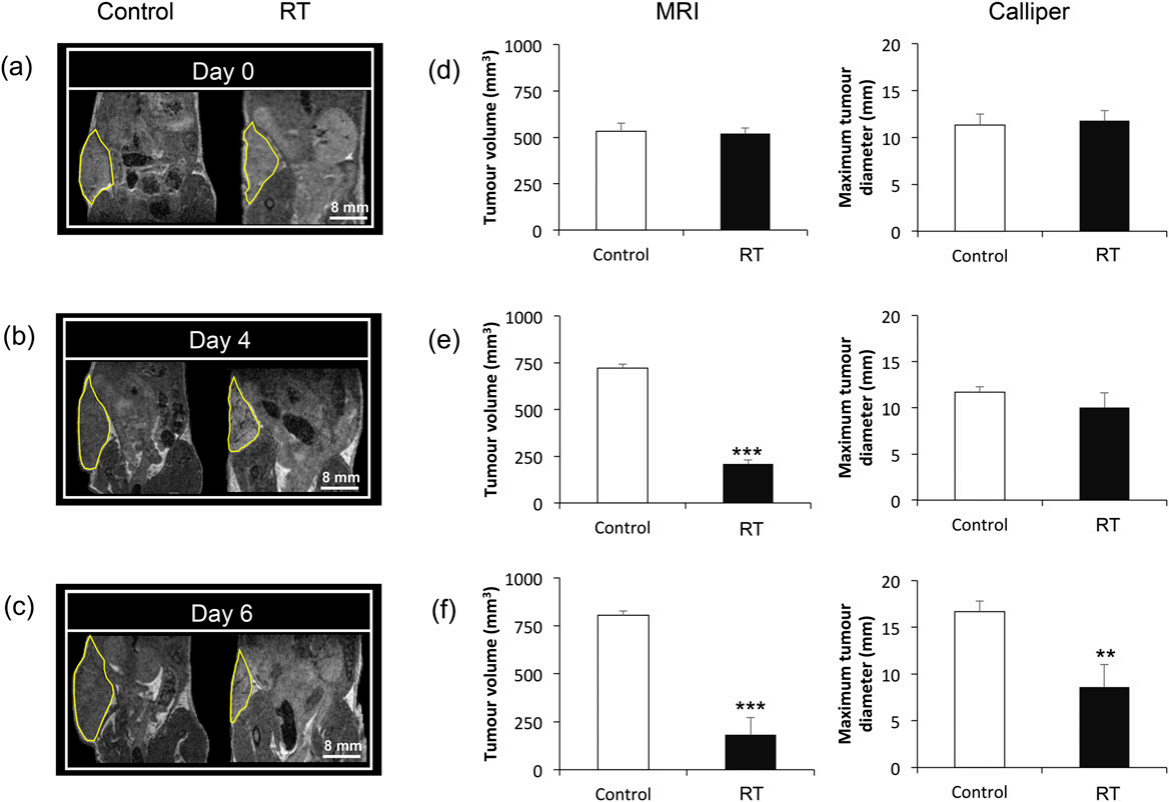
Response of SK-N-SH tumours to radiotherapy (RT) measured using MRI and callipers: representative MR images of control (left) and RT-treated (right) tumours at (a) Day 0 (before treatment), (b) 4 days and (c) 6 days after RT. Tumours are outlined in yellow. Bar charts show tumour volume measured by MRI and maximum tumour diameter measured by calliper at (d) Day 0 (before treatment), (e) 4 days and (f) 6 days after RT (Mean ± standard deviation), ***p < 0.001 and **p < 0.01, RT *vs* control group.

Serial MRI was performed to assess tumour response to RT and compared with calliper measurements. On Day 0 (before treatment), the tumour volume, as assessed by MRI or calliper measurements, was similar in the control and RT groups (Figure 4a,d). At 4 and 6 days following treatment, MR images showed a marked decrease in the tumour volume in the RT *vs* control groups (Figure 4b,c). Interestingly, it was found that the change in tumour size at 4 days was not detected by calliper measurements (*p* = 0.14) but was apparent using MRI assessment (*p* < 0.001) (Figure 4e). The treatment effect became observable with calliper assessment only from Day 6 (*p* < 0.01) (Figure 4f).

**Figure 5.**
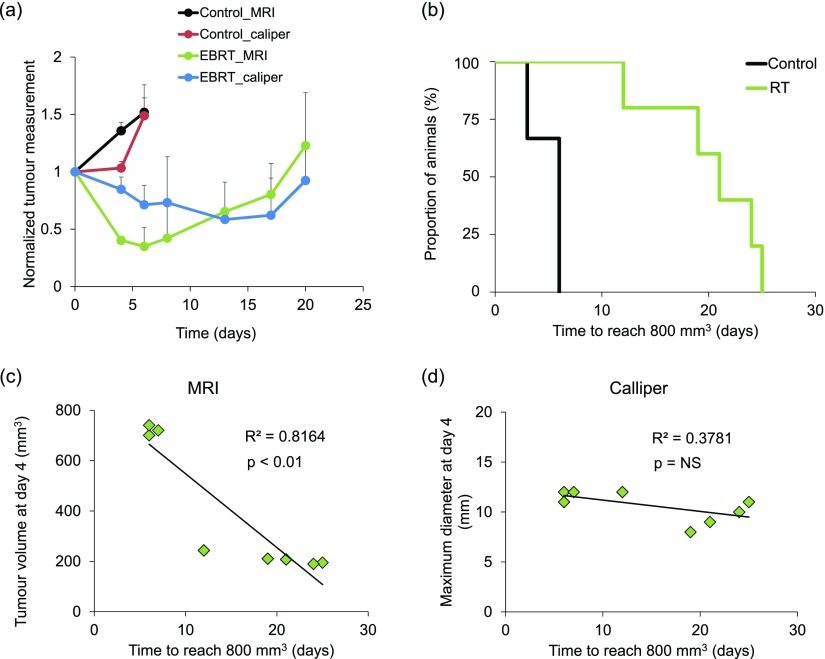
Correlation between tumour volume and survival based on MRI *vs* calliper measurements: (a) tumour volume (relative to pre-treatment) of control and radiotherapy (RT)-treated mice using MRI *vs* calliper measurements (mean ± standard deviation). (b) Kaplan–Meier curves showing survival of control *vs* RT-treated mice (animals were euthanized when tumours reached 800 mm^3^), *p* < 0.01. Correlation between survival time and tumour volume/size as measured by (c) MRI and (d) calliper. EBRT, external beam RT; NS, not significant.

Serial MRI and calliper measurements of tumour size were performed to assess the ability of the two methods to detect tumour progression. After an initial decrease in tumour volume, tumour regrowth was observed from 8 days after RT with MRI (Figure 5a). However, expansion of tumour volume was not detected by calliper assessment until Day 17. There was a significant increase in survival time in the RT group compared with the control group (*p* < 0.01) (Figure 5b). These survival data were used to evaluate the ability of both methods (MRI and calliper) to predict the overall survival at an early time point (4 days) after RT (Figure 5c,d). There was no correlation between maximum diameter measured by calliper and survival at 4 days (*R*^2^ = 0.38; *p* = 0.11) (Figure 5d). However, the tumour volume derived from MR images at 4 days did correlate strongly with survival (*R*^2^ = 0.82 and *p* < 0.01) (Figure 5c).

## DISCUSSION

The SK-N-SH cell line is a preclinical model of human neuroblastoma that has been used extensively to generate xenograft tumours in mice.^7^ However, the implanted SK-N-SH cells do not form firm, well-defined masses. This presents a problem when treating tumours with external radiation, as it is difficult to define the extent of tumour infiltration, potentially increasing the risk of irradiating adjacent normal tissues or missing tumours. This is relevant to high-risk neuroblastoma in children, since it forms poorly marginated, infiltrative tumours that often lie in close proximity to radiosensitive organs such as the intra-abdominal viscera, lungs and spinal cord. To address this problem, we developed an MR-guided RT protocol using an in-house consecutive MRI-SARRP protocol. A purpose-built, plastic cradle that allows MRI and CBCT co-registration was designed and constructed. The cradle facilitated MATLAB^®^ co-registration of MR and CBCT images, allowing the RT target volume to be planned directly on the anatomical MR image. Our results concur with those of Bolcaen et al,^8^ who showed that MRI was superior to CBCT for the delineation of the tumour volume prior to RT in a preclinical brain tumour model. Furthermore, this approach led to a significant normal tissue dose decrease during RT.^8^

In the present study, measurement by calliper, a common method for the evaluation of xenograft size, was not sufficiently sensitive to detect early tumour regrowth and measurements at early time points did not correlate with survival time. This is partly owing to the physical characteristics of SK-N-SH xenografts, as they from flattened, poorly defined tumours in nude mice, making them difficult to measure by calliper (Figure 2b). In contrast, anatomical MRI assessment of tumour volume provided insight into the early effects of treatment and correlated with length of survival time. The utility of other imaging modalities to detect tumour response has been investigated. For example, Valentiner et al^9^ demonstrated the advantages of using 18F-fluorothymidine (FLT) positron emission tomography (PET) compared with 18F-fluorodeoxyglucose PET for tumour detection in the SK-N-SH model. Robust methods to plan and assess RT efficacy in this preclinical model is vitally important for the evaluation of new therapeutic approaches: for example, protocols that combine RT with chemotherapy and radionuclide therapy.^10^

## CONCLUSION

An MRI-guided RT protocol for the treatment of a widely used human neuroblastoma model has been developed. MRI provides accurate monitoring of tumour volume, thereby facilitating the early detection of the RT treatment effect and its predictive value on overall survival.

## FUNDING

This work was supported through the CR-UK/MRC Oxford Institute for Radiation Oncology and the CR-UK/EPSRC Oxford Cancer Imaging Centre. MNG is supported by the NIHR University College London Hospitals Biomedical Research Centre.
